# Transposable elements, mRNA expression level and strand-specificity of small RNAs are associated with non-additive inheritance of gene expression in hybrid plants

**DOI:** 10.1186/s12870-015-0549-7

**Published:** 2015-07-03

**Authors:** Qing Li, Ying Li, Stephen P. Moose, Matthew E. Hudson

**Affiliations:** Department of Crop Sciences, University of Illinois at Urbana-Champaign, Urbana, IL 61801 USA

**Keywords:** Hybrid, Gene expression inheritance, Small RNA, Transposon

## Abstract

**Background:**

Gene expression inheritance patterns in Arabidopsis hybrid plants were investigated for correlation with the presence of transposable elements (TEs) and small RNA profile.

**Results:**

The presence of TEs in a gene and the expression of small RNA matching a gene were both found to be associated with non-additive mRNA inheritance patterns in hybrids. Expression levels below mid-parent values in the hybrids were associated with low mRNA expression in parents, with the presence of small RNA from both strands, and with the presence of TEs. High-parent dominance of mRNA levels was found to be associated with high parental mRNA expression levels, the absence of TEs, and for some genes, with small RNA fragments that are predominantly from the sense strand. These small RNAs exhibit a broader size distribution than siRNA and reduced nucleotide end bias, which are consistent with an origin from degraded mRNA. Thus, increased as well as decreased gene expression in hybrids relative to the parental mean is associated with gene expression levels, TE presence and small RNA fragments with differing characteristics.

**Conclusions:**

The data presented here is consistent with a role for differential mRNA decay kinetics as one mechanism contributing to high-parent dominance in gene expression. Our evidence is also consistent with *trans* repression by siRNA and TEs as the cause of low-parent dominance.

**Electronic supplementary material:**

The online version of this article (doi:10.1186/s12870-015-0549-7) contains supplementary material, which is available to authorized users.

## Background

Hybrid plants can show enhanced growth rate, novel phenotypes or adaptive advantages in diverse environments. They may even have enhanced potential to evolve into new species [1, 2]. The unique biology of hybrids has led to the proposal of many hypothetical molecular, genetic and epigenetic mechanisms to explain the differences between progeny and progenitors [3–9]. One intriguing hypothesis is that variation in gene expression is a driver of hybrid biology. Expression variation has been proposed as a driver of phenotypic changes, species evolution and domestication [10–13]. Gene expression levels in hybrids can vary in comparison to the levels in either parent, usually classified into additive/mid-parent values (MP) and several non-additive categories, such as low-parent dominance (LP) or high-parent dominance (HP), although expression outside the range of the two parents is rare [14]. The relative proportion of non-additive inheritance usually varies across species, crosses and tissues [14–21]. While the underlying mechanisms regulating gene expression in hybrids remain to be discovered, small RNA and transposable elements (TEs) have been proposed as likely agents that may trigger non-additive inheritance [22, 23].

TEs were discovered by McClintock and proposed as regulators of gene expression [24]. Recent studies suggest that TEs can mediate genome-wide rewiring of gene regulatory networks [25]. There are many possible ways that a TE could affect gene expression [26], including disrupting gene integrity by inserting into genes, creating or disrupting direct *cis-*regulatory elements, and capturing and duplicating genes or gene fragments [27, 28]. The proteins encoded by TEs and their relatives can also affect gene expression [29]. Another way TEs may modify gene expression is to sensitize a gene to be under epigenetic control. Since TEs are usually epigenetically silenced [30, 31], silencing can spread from TEs to closely linked genes [32]. This spread could cause reduced expression of nearby genes [33, 34]. The regulatory effect of TEs on gene expression is rather stable, can persist through hybridization and be transmitted across generations [35].

Small RNA is composed mostly of miRNA and small interfering RNA (siRNA). The role of siRNA is generally considered to be the silencing of TEs and genes, which occurs largely by RNA-directed DNA methylation or post-transcriptional pathways [36, 37]. Both siRNA and TEs can regulate gene expression. For instance, in the case of paramutation, where gene expression levels in the progeny are similar to one parent and deviate from the mid-parent value, siRNA has been proposed to be the control mechanism [38]. Similarly, insertion of a TE in a gene or promoter can alter expression patterns or levels. Given the role of TEs and siRNA in gene expression regulation, and the relative speed with which plant genomes diverge in both TEs and siRNA content, we investigated the observed diversity of gene expression levels in a hybrid system for links to either TE or siRNA or both. In our study, we found a number of such links, and we also found a correlation between short sense strand RNA fragments, high levels of gene expression and HP inheritance.

## Results

### Inheritance patterns of mRNA and genic small RNA in Arabidopsis hybrids

To study how mRNA and genic small RNA levels are inherited in hybrids, we sequenced mRNA and small RNA from four genotypes, Col, Ler and their reciprocal hybrids Col × Ler (Col maternal) and Ler × Col (Ler maternal) from leaf tissues (Additional file 1). We used four pooled leaves from a single plant to prepare each total RNA sample, with four plants producing four biological replicate samples. This was done to preserve the variation in mRNA and small RNA from a single individual, rather than using a mixture of different plants which may vary for small RNA and mRNA levels. We reasoned that the use of a single plant per replicate would facilitate the study of dynamic co-regulation between small RNA and mRNA levels. We obtained 10–17 million mapped mRNA reads and 1–2 million mapped small RNA reads for each library (Additional file 2). At this sequencing depth, a total of 17,121 and 2,106 protein-coding genes were found to uniquely match sequenced mRNA and genic small RNA, respectively; 434 genes were found with both mRNA and genic small RNA (Additional file 3).

At FDR < 0.05, we found 5,858 genes with differentially expressed mRNA and 1,093 genes with differentially expressed genic small RNA among Col, Ler and the hybrids (Table 1). These differentially expressed genes could be assigned into seven groups based on the comparison of the expression level in hybrids and their two parents (Table 1). For mRNA, the expression level of most of the genes (3,654 or 62.4 %) was additive (MP). About 31 % of the genes showed non-additive expression levels in the hybrids similar to one of the two parents, either HP or LP. We also detected several genes that showed expression levels between the MP value and LP value (LPMP). There were very few cases (37 genes, or 0.7 %) where the hybrid expression levels were between the MP value and HP value (HPMP) or outside the parental range (AHP or BLP). Similar to mRNA, most of the genes (54.3 %) with genic small RNA showed small RNA levels inherited additively in hybrids. About 37.2 % of these genes showed LPMP and LP inheritance of small RNA levels (Table 1), similar to previous findings in Arabidopsis and other species [22, 39, 40]. Genes with genic small RNA levels outside the parental range were rare, as in the case of mRNA. We looked at gene ontology for the genes with different inheritance patterns of mRNA, and found that genes with MP, LPMP and LP inheritance patterns were mostly enriched in the categories of response to stress, protein amino acid phosphorylation and nucleotide binding activity. The genes with HP inheritance patterns were mainly enriched in photosynthesis and ribosome structural composition (Additional file 4). Because of the very low number of genes with AHP, HPMP, and BLP patterns, the following analysis will focus on the 5,618 genes with HP, MP, LPMP and LP inheritance patterns for mRNA levels.

**Table 1:** Number of genes observed with additive and non-additive inheritance patterns of genic small RNA and mRNA in Arabidopsis hybrids

Inheritance pattern	Gene count		Percentage (%)	
	mRNA	Small RNA	mRNA	Small RNA
AHP	9	0	0.2	0
HP	827	65	14.1	6
HPMP	6	7	0.1	0.6
MP	3,654	594	62.4	54.3
LPMP	158	215	2.7	19.7
LP	979	191	16.7	17.5
BLP	22	5	0.4	0.5
UC	203	16	3.5	1.5
Total	5,858	1,093	100	100

*AHP* above high-parent, *BLP* below low-parent, *HP* high-parent, *HPMP* between mid-parent and high-parent, *LP* low-parent, *LPMP* between mid-parent and low-parent, *MP* mid-parent, *UC* unclassified (Hybrid is significantly different from MP, but not significant from both HP and LP)

### Small RNA and TEs correlate with different mRNA inheritance patterns in hybrids

**Fig. 1:**
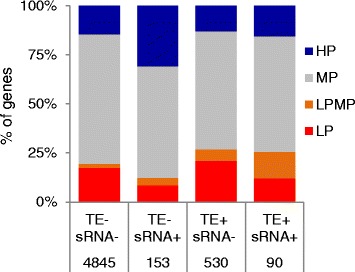
Small RNA (sRNA) and TEs are associated with mRNA inheritance patterns in hybrids. The percentage of genes differentially expressed between the two parents with different inheritance patterns (HP, high-parent; LP, low-parent; LPMP, between low-parent and mid-parent; MP, mid-parent) are plotted on the y axis for each of four TE/sRNA categories: TE-, absence of any TEs; TE+, presence of one or more TEs; sRNA-, absence of small RNA; sRNA+, presence of small RNA. The number of genes in each of the four TE/sRNA categories is given below each category

To study whether TEs and the production of small RNAs might influence the inheritance patterns of gene expression, mRNA inheritance in hybrids was compared among four classes of genes: genes lacking TEs and small RNAs; genes with one or more TEs within the gene boundaries but no small RNAs; genes with small RNAs but no TEs; and genes with both one or more TEs and small RNAs (Fig. 1). Out of the 5,618 differentially expressed genes, ~15 % of them have either small RNA matching the gene, a TE within the gene, or both. Compared to genes that are without TEs or small RNA, this set of genes shows a higher proportion of non-additive gene inheritance patterns (Fig. 1). More genes with small RNA but not TEs show a HP pattern; however when TEs are present, more LPMP and LP genes are found. Interestingly, genes with both TEs and small RNA show similar proportions of inheritance patterns to genes that only have TEs. The increase in LPMP and LP inheritance patterns was most obvious when the TE was within the gene boundaries, and decayed rapidly when a TE was located nearby, upstream or downstream of the gene (Additional file 5).

### Presence of small RNA, TEs and mRNA inheritance patterns correlate with mRNA expression levels in parents

**Fig. 2:**
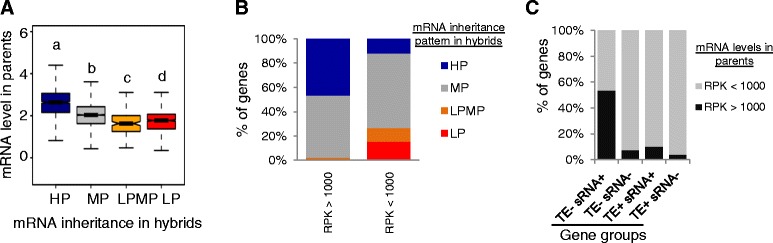
The expression level of genes with different inheritance in hybrids, and genes with TEs or sRNA. **a** mRNA inheritance in hybrids is related to mRNA abundance (expression level) in the parents. The letters a, b, c and d above each box indicate the mRNA level is significantly different between the genes in each group and all three other groups with different mRNA inheritance patterns based on a Wilcoxon rank-sum test. **b** mRNA inheritance for genes with different expression levels in parents. **c** Genes with small RNA but no TEs (TE- sRNA+) tend to be highly expressed genes (RPK > 1000). RPK = normalized mRNA read counts per kb. HP, high-parent; LP, low-parent; LPMP, between LP and MP; MP, mid-parent; sRNA: small RNA

We studied whether mRNA expression level in parents was associated with mRNA inheritance patterns in hybrids. We found that genes with different mRNA inheritance patterns show different mRNA levels in parents. While genes with HP inheritance patterns show the highest median expression levels, genes with LPMP and LP patterns show relatively low median expression levels. The lowest mRNA abundance of all was observed for genes with LPMP inheritance (Fig. 2a). This pattern was further illustrated by comparing mRNA inheritance patterns for genes with different mRNA abundance in parents. While highly expressed genes (RPK > 1000) were often associated with HP inheritance in hybrids, genes with lower levels of mRNA expression (RPK < 1000) were frequently associated with LP inheritance (Fig. 2b). Thus, highly-expressed genes tend to show HP inheritance patterns, and genes with lower expression levels are more likely to show LP inheritance patterns. As expected from the observations that HP-inherited genes are associated with small RNAs but lack TEs, this class of genes are enriched in highly-expressed genes (Fig. 2c).

### Small RNA that correlates with high mRNA expression levels in parents and HP inheritance in hybrids is predominantly from sense strand

**Fig. 3:**
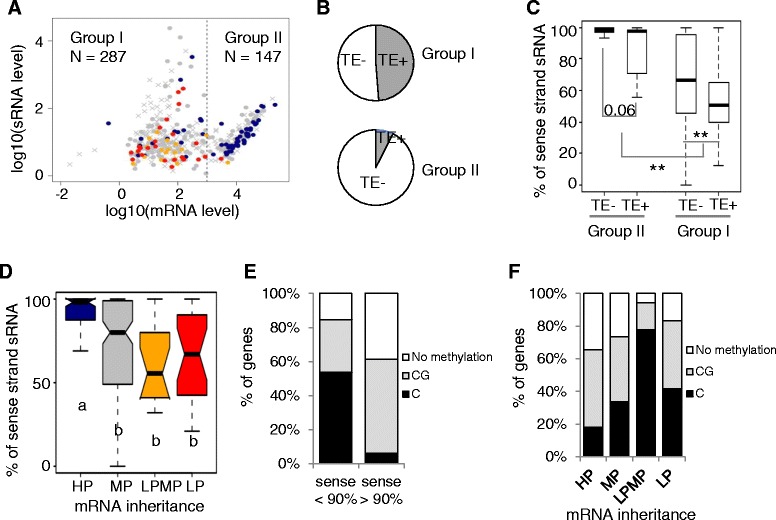
Association between mRNA level and sRNA level depends on the strand specificity of the sRNA. **a** Plot of sRNA abundance against mRNA expression level. The grey “x” represents genes that were not differentially expressed. Dots of different colors represent differentially expressed genes with different mRNA inheritance patterns. Blue, HP; grey, MP; orange, LPMP; red, LP. Genes were divided into two groups based on their mRNA abundance, “group I” (RPK < 1000) and “group II” (RPK > 1000). Group II genes showed significant positive correlation between sRNA level and mRNA level. **b** Percentage of genes with gene body TEs in “group I” and “group II” genes. **c** Percentage of sense strand sRNA for genes in “group I” and “group II”. **,*P* < 0.01. **d** mRNA inheritance is correlated with sRNA strand-specificity. High parent inherited genes (a) show statistically significant enrichment for sense-strand specific sRNA, other inheritance patterns (b) show roughly equal levels of sRNA from the two strands. **e** Genes with more than 90 % of small RNA from the sense strand (sense > 90 %) show more CG methylation in the gene body. **f** mRNA inheritance pattern and DNA methylation pattern. HP, high-parent; LP, low-parent; LPMP, between LP and MP; MP, mid-parent; sRNA: small RNA

The association of TEs and small RNA with reduced gene expression has been well documented previously, but it was unexpected that the presence of small RNA (without TEs) was found to be associated with increased gene expression. To provide further insight, we studied the relationship between genic small RNA and mRNA levels using the two parental genotypes, Col and Ler. We found that small RNA level was positively correlated with mRNA level, but only when mRNA expression was above a clearly defined threshold of around 1,000 normalized reads (Spearman Correlation Coefficient > 0.75, Fig. 3a, Additional file 6). Interestingly, both the presence of TEs and the strand from which small RNA was detected were strongly differentiated between the genes above and below this expression threshold. The genes with high mRNA levels, as expected, were much less likely to have TEs than genes with low mRNA levels (Fig. 3b, 8 % vs 49 %, χ^2^ test, *P* < < 0.001). Moreover, small RNA of the highly expressed genes predominantly or exclusively matched the sense strand (i.e. the mRNA itself), while sense and antisense strands were roughly equally expressed from genes where mRNA was expressed at lower levels (Fig. 3c). We studied how small RNA strandedness in parents correlates with mRNA expression patterns in hybrids (Fig. 3d). We found more sense strand small RNA (sssRNA) in the genes with HP inheritance, and more small RNA from both strands in the genes with LPMP and LP inheritance. Significantly, this distribution of sssRNA levels closely resembled that of mRNA levels (compare Fig. 2a and Fig. 3d).

DNA methylation has been suggested to be associated with reduced gene expression, and the presence of gene body methylation is linked and correlated with the presence of transposons [41]. However, gene-body methylation in the CG sequence context is frequently associated with high mRNA expression level [42–45]. Since we observed an association between sssRNA and high mRNA abundance, we were interested to see how sssRNA correlates with DNA methylation. Using a recently published dataset on the Arabidopsis methylome [42], we investigated the possibility that different DNA methylation patterns are associated with sssRNA. We found that genes with small RNA derived predominantly from the sense strand correlate with genes with CG methylation or no methylation. On the other hand, genes with small RNA from both strands tend to have methylation in all sequence contexts (Fig. 3e). We also observed that genes with high-parent inheritance were more likely to lack methylation, and genes with LPMP inheritance are very likely to be methylated (Fig. 3f). While sssRNA is associated with high-parent inheritance pattern in hybrids (Fig. 3d), such an association was not observed for CG methylation (Fig. 3f).

### Observed patterns are replicated in a second cross

In order to investigate the possibility that our results were in some way specific to our experimental conditions, we used the data of Shen et al. [46] to investigate the relationships in a second experimental hybrid system. Shen et al. used different Arabidopsis accessions (Ler and C24 versus Col and Ler in this study), different tissues (seedling versus mature leaves), and a different sequencing platform (Illumina GAII versus HiSeq 2000). Similar to the results in our dataset, we found that small RNA levels were positively associated with mRNA level when mRNA abundance was above a threshold level (Additional file 7A). Those highly expressed genes more frequently had sssRNA, and were less likely to have TEs (Additional file 7B, C), while genes expressed at low levels were more likely to have both sense and antisense small RNA, and TEs (Additional file 7B, C). We also observed association between sssRNA and high parent-inheritance (Additional file 7D), but were not able to statistically evaluate this result because of limited replication in the second dataset. Also, we found that genes with predominantly sssRNA were frequently associated with CG methylation (Additional file 7E). Similar to the results in the Ler x Col cross, genes with HP-like inheritance patterns had reduced levels of DNA methylation (Additional file 7F).

### The sssRNAs likely arise from mRNA degradation

**Fig. 4:**
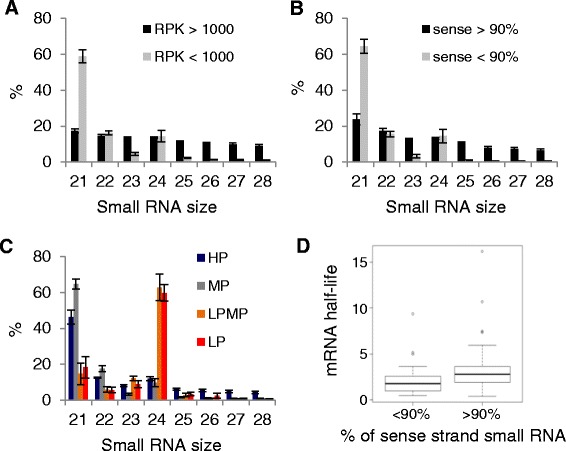
Sense strand sRNAs are not canonical small interfering RNA. **a** Comparison of sRNA size distribution for highly expressed (RPK > 1000) and lower expressed genes (RPK < 1000). **b** Distribution of sRNA size for genes with predominantly (>90 %) sssRNA and genes with less than 90 % of sRNA from the sense strand. **c** Distribution of sRNA size for genes with different mRNA inheritance patterns. **d** mRNA half-life for genes with different percentages of sssRNA. Error bars represent the standard deviation of four biological replicates. RPK = normalized mRNA read counts per kb. HP, high-parent; LP, low-parent; LPMP, between LP and MP; MP, mid-parent; sRNA: small RNA

In order to determine the molecular origin of the sssRNAs that are correlated with high parent expression, we first looked at the size distribution of these species to assess whether they fit the canonical 21-24 nt size range typical of processing by DICER [47]. Fig. 4a-c show the size distribution for small RNAs in the range 21-28 nt, for high and low expressed mRNAs (according to the threshold in Fig. 3a), loci producing predominantly sense strand species versus mixed species, and the four inheritance classes for each locus. Both highly-expressed genes and genes with mostly sssRNAs produce similar proportions of RNAs in each size class with a slight progressive bias to shorter RNAs, a distribution consistent with products of mRNA degradation instead of processing by DICER. The genes that show HP inheritance patterns have slightly more 25-28 nt small RNA but 21-24 nt small RNAs are still the dominant small RNA species. While the genes that show LP inheritance have predominantly 24 nt small RNA indicative of siRNAs, genes with HP or MP inheritance have a high proportion of 21 nt species (Fig. 4c).

Additional observations indicate that the sssRNAs are likely associated with mRNA degradation. Firstly, degradation intermediates, along with regulatory small RNAs, are captured in small RNA libraries because they are in the selected size range and possess a free 5’PO_4_^−^ group capable of adaptor ligation. Secondly, the sssRNAs do not exhibit the marked bias to 5’ terminal uridine (miRNAs) or adenosine nucleotides (siRNAs) that is preferred for ARGONAUTE binding (Additional file 8). Thirdly, we investigated whether sssRNA correlated with mRNA half-life [48]. The mRNAs associated with genes producing high levels of sssRNA showed a tendency to have longer half lives than other genes (Fig. 4d), linking the production of these species with the mechanisms of RNA degradation.

## Discussion

### TEs and small RNA are associated with high probability of non-additive mRNA inheritance in hybrids

Following hybridization, differentially expressed genes between parents could follow the mean of the two parents (MP, additive expression), or deviate from MP (non-additive expression, eg. HP or LP). The proportion of genes showing additive and non-additive expression usually varies substantially between studies [49], and this has been widely attributed to the different assay platforms, species, crosses and tissues. There are a number of possible regulatory mechanisms that could lead to non-additive gene expression, which complicates prediction of the phenotypic consequences from hybridization. Here, we present that integrated knowledge of nearby TEs, mRNA expression level, and small RNA strandedness in parents serve as indicators of the direction of deviation from additive expression.

For genes that had both small RNA and TEs, the gene expression levels are generally low and the small RNAs possess the expected properties of siRNAs. These genes are often associated with a low-parent inheritance pattern, which is consistent with a role for siRNA in gene silencing in hybrid plants. We propose that siRNAs and TEs are therefore causally related to LP and LPMP inheritance. However, for genes with detectable small RNA but no TE, the gene expression levels are often very high, and small RNA sequencing identifies predominantly or exclusively sense-strand derived fragments with high proportions of 25-28 nt RNAs and little noticeable 5’ nucleotide bias. These features are correlated with HP inheritance in the hybrids. The fact that small RNA from different strands is associated with contrasting non-additive inheritance patterns suggests that small RNA strandedness should be considered in future studies on small RNA and gene expression pattern. We should note that while we observed strong correlations in this study, these are significant associations not qualitative relationships. Many exceptions exist, such as highly expressed genes that contain TEs, or additively expressed genes that produce siRNA. It is therefore possible that if these phenomena are causative of non-additive gene expression, they may not be the only mechanisms regulating non-additive expression in hybrids.

### Mechanistic implications of sssRNAs and high-parent inheritance

Despite a long-known association between siRNA, DNA methylation and low levels of mRNA expression, it has previously been described that genes with body methylation can show higher expression than genes that are unmethylated [44, 50]. One explanation is that CG (as opposed to all three types (CG, CHG and CHH, H = A, C, or T) of methylation) is associated with higher expression levels [42]. Examples where siRNA level is positively correlated with mRNA level in hybrids have been described before [39, 40]. Here, at the genome-wide scale in Arabidopsis, we found that small RNA sequencing can also identify small RNAs whose accumulation is positively correlated with mRNA level (Fig. 3a), occurs in the absence of TEs (Fig. 3b) and are derived predominantly from the sense strand (Fig. 3c). These sssRNAs are most likely created by endonuclease activity on mRNA from highly expressed genes. Supporting this conclusion, the sssRNAs exhibited a wider size distribution and lacked the 5’ terminal nucleotide bias of 21-24 nt for miRNAs or siRNAs (Fig. 4, Additional file 8). Also these sssRNAs are derived from sequence throughout the transcript (Additional file 9), making it unlikely that they are products of the DCL family enzymes.

Importantly, we observe a large number of highly-expressed genes that produce no detectable small RNA. Thus, sssRNAs do not appear to be a general outcome from mRNA degradation. One explanation for this difference is that not all highly expressed genes may produce sufficiently stable 21-24 nt degradation intermediates to be detected by the RNA-adaptor ligation approaches that are used to generate small RNA libraries. We also considered the possibility that ARGONAUTE binding could preferentially stabilize the subset of mRNA degradation products with 5’ U or A terminal nucleotides, but did not find evidence supporting this hypothesis (Additional file 8). Additionally, these sssRNAs were from genes that tend to have longer half-lives, which is in contrast to the expected abundance of mRNAs derived from endonuclease degradation through a common pool of enzymes and intermediates (see below). We therefore have no definitive evidence for the mechanism by which sssRNAs were produced.

We observed a correlation between HP inherited mRNA expression level in hybrid offspring and the presence of sssRNAs matching the HP inherited mRNAs. We propose three possible mechanisms by which sssRNA might be linked to a mechanism for HP inheritance, each of which could be tested with future work.

One explanation of this correlation could be that sssRNAs may functionally contribute to trans-acting regulatory mechanisms that, in turn, stimulate higher expression of both loci in a hybrid diploid. This could occur in a manner analogous, but opposite in outcome, to the repressive effects of siRNA and TEs that mediate LP or LPMP expression. Interaction of sssRNAs with other factors that either activate or repress gene transcription would then mediate this effect. However, this speculative mechanism would require the invocation of a previously undiscovered, sssRNA-mediated mechanism for transcriptional activation.

Another explanation is that sssRNAs could be indicative of different degradation rates of mRNAs, and that mRNA degradation is therefore implicated in the mechanism of HP inheritance. We expect that, once free of the 5’ cap and 3’ poly (A) tail or cleaved by miRNA-targeted action, mRNAs are degraded by enzymes, largely exonucleases, that are not sequence specific. On this basis, all mRNA in the process of degradation would be part of a common pool, and thus, for two mRNAs with the same overall level of accumulation but different half-lives, degradation intermediates of the shorter-lived mRNA would be expected to be more common, since this transcript will have a higher rate of synthesis and thus more intermediates at each stage of degradation. We would also expect oligomer intermediates such as sssRNA to be biased towards certain sites, which are furthest from the site unprotected from exonuclease action by cleavage or tail/cap loss. Since we observe more sssRNA for longer-lived mRNAs, and sssRNA throughout the transcript, our results are not consistent with sssRNA being the expected degradation intermediates of mRNAs. One possible explanation is that certain mRNAs with longer half-lives are degraded differently, producing degradation intermediates with different positional bias and longer half-lives. This could be a result of varying affinities of the degradation machinery for different mRNAs, or be the consequence of a property of the mRNAs (for example, secondary structure or ribosome occupancy). Either way, this explanation requires different mRNAs to show different degradation rates and/or fragment sizes after degradation has been initiated by miRNA-directed cleavage, loss of poly (A) tail or loss of 5’ cap. In hybrids, mRNA degradation could therefore act differentially on a subset of highly expressed genes, leading to HP inheritance of those genes.

A third, related possibility is that the sssRNA are an indication of degradation pathways becoming overloaded under certain conditions in hybrids, leading to HP inheritance through reduced degradation rates. A link was recently reported between cytoplasmic mRNA decay and suppression of post-transcriptional gene silencing (PTGS) [51]. Disruption of RNA decay increases PTGS, as evidenced by the increased production of siRNAs from protein coding transcripts (named ct-siRNAs). These ct-siRNA were also shown to be associated with highly expressed genes, leading to the interpretation that when RNA decay pathways are disrupted by genetic mutation or overloaded by high-level expression of transgenes or endogenous genes, aberrant transcripts are channeled into the PTGS pathway. Although our evidence does not support sssRNAs as being produced via PTGS, our observations are consistent with the idea that when parental gene expression levels are very high, they may overload cellular capacity for mRNA degradation under some conditions. Increased levels of RNA production and degradation in hybrids, as a general consequence of hybrid vigor or of epigenetically activated transcription, could lead to a global “degradation overload” effect on gene expression inheritance, one which may be more prevalent among genes with highly stable mRNAs. A subtly different interpretation would be that since our data is derived from bulk mRNA extraction from leaf tissue, it is possible that mRNA degradation is overloaded in some cell types only, and both HP inheritance and sssRNA are a consequence of high level, cell-specific expression of certain genes in hybrids, in a subset of the cells investigated. If mRNA degradation is indeed limiting in some cell types, sssRNA would be prevalent for the genes highly expressed in these cells, and these mRNAs also tend to have longer than average half lives.

## Conclusions

In summary, we describe significant associations between mRNA expression levels, small RNA fragments, TEs and non-additive gene expression in hybrids. We show evidence that sssRNA is associated with high mRNA expression in parents of a cross and with HP inheritance in hybrids. We also show that LP and LPMP inheritance are correlated with low mRNA expression in parents, with small RNA from both the sense and antisense strands, with the presence of TEs and with DNA methylation in all sequence contexts. Thus, known mechanisms of transcriptional silencing may account for LP and LPMP inheritance patterns via *trans* repression of genes by siRNA inherited from one parent, much of which is initiated by the presence of TEs within gene bodies. For HP inheritance, a challenging phenomenon to interpret using currently understood mechanisms of gene expression control, we suggest that overloaded mRNA decay may be involved in mediating HP inheritance patterns for at least some highly-expressed genes.

## Methods

### Plant materials

Four genotypes were used in this study, namely Columbia (Col), Landsberg *erecta* (Ler), and their reciprocal hybrids Col × Ler (Col is the maternal genotype), Ler × Col (Ler is the maternal genotype). The source of Col and Ler has been described before [22]. The hybrids were obtained by manually crossing Col and Ler. Inbreds Col and Ler were also manually selfed to minimize the effect caused by manual crossing across the four genotypes. Seeds from the same cross were collected and pooled together.

Seeds were sterilized, planted on MS medium, stratified at 4 °C and germinated at 20 °C under 24 h cycles, 16 h of light and 8 h of darkness. Plants were then transplanted into soil (sunshine mix : perlite : vermiculite = 2 : 1 : 1) and grown under same conditions until bolting. The four youngest mature leaves (defined as leaf with petiole) from a single plant were harvested, pooled and viewed as one biological replicate. Four biological replicates were collected for each of the four genotypes. All materials were collected at around the same time every day to minimize effect of circadian clock, which has been shown to affect gene expression [52]. Some of the remaining leaves were harvested to extract DNA, which was used to confirm the genotype with a genetic marker (nga106) that is polymorphic between Col and Ler [22].

### Library preparation and sequencing

Total RNA was prepared from a single plant using TRIzol according to manufacturer’s instructions (Invitrogen). As suggested by the Illumina protocol, 1 μg of total RNA was used to prepare each mRNA sequencing library using the TruSeq RNA sequencing library preparation kit (Illumina). For small RNA library preparation, small RNA with sizes between 10-nt and 50-nt was first isolated from 15 μg of total RNA using 15 % urea-PAGE (Invitrogen). The purified small RNA was then used to prepare a sequencing library according to the instructions supplied with the TruSeq small RNA library kit (Illumina). Both RNA and small RNA libraries were barcoded and pooled. Sequencing was performed on Illumina HiSeq 2000 instruments, single-end reads of 100-nt were generated for the mRNA libraries and 40-nt for small RNA libraries. On average, 15.7 and 8.1 million reads were obtained for each RNA and small RNA library, respectively.

### Mapping onto genome and summarizing read counts per gene

The RNA reads were mapped onto Arabidopsis Col genome version 10 (TAIR10) using tophat allowing at most 2 mismatches [53]. Reads mapping to more than one genome location were discarded. HTSeq [54] was used to summarize read counts for each gene model. To make reads comparable across genotypes and across genes, the raw read counts per gene in each library was first normalized using the method implemented in DESeq [55], and were further normalized to gene size.

For small RNA, FASTX [56] was used to trim 3’ adapter, control quality (allowing 1-nt to have quality score below 30), and select small RNA size (21 nt-24 nt). Reads that mapped to miRNA, tRNA and rRNA were further removed, leaving only the siRNA [22]. Non-redundant reads from all 24 libraries were then pooled and mapped onto the TAIR10 genome using bowtie [57]. When allowing 0 mismatches, only 36 % ~ 56 % of the sequencing reads could be mapped. The mapping rate increased to 59 % ~ 72 % when allowing 1 mismatch. To ensure only high quality reads were used; we only kept reads that were perfectly mapped to the genome. This lead to the usage of on average 1.6 million reads (0.4 million distinct siRNA signature) per library. For reads that were mapped to more than 1 genome location, the read counts for each location were normalized to the total number of chromosomal locations. Small RNA levels from each gene were calculated based on the gene model boundaries derived from the GFF file. Read counts for each gene were summarized using an in-house Perl script. Read counts for small RNA from each strand were also summarized to get the percentage of sssRNA.

### Differential expression analysis and classification of inheritance in hybrids

An R package, DESeq [55] was used to identify differentially expressed genes for each pair-wise comparison. Genes without ≥ 20 reads in at least 4 libraries were discarded from the analysis. False discovery rate (FDR) correction was used to correct for multiple comparisons and genes with FDR < 0.05 were called significantly differentially expressed. Using these criteria, only one out of the 17,121 genes was differentially expressed between the two hybrids. This is AT5G47230, which encodes a member of the ERF (ethylene response factor) subfamily B-3 of ERF/AP2 transcription factor family (ATERF-5). Since parent-of-origin effects were small, the two hybrids were combined together to assign gene inheritance patterns, thus increasing statistical power. Based on the comparison of observed hybrid values and high-parent (HP), low-parent (LP) and mid-parent (MP) values, genes were classified into 7 categories according to Stupar et al. [14]. Briefly, a gene was called MP if the hybrids were not significantly different from MP at FDR < 0.05. For the genes where the hybrids were significantly different from MP, they would be called LP if hybrid expression levels were not different from LP but different from HP, and called HP if *vice versa*. If hybrid expression levels were different from all three of MP, HP and LP, the d/a value (“d” is defined as “F1 - (HP + LP) / 2”, and “a” is defined as “(HP - LP)/2”) was further used to group genes into AHP (above HP) if d/a was greater than 1, HPMP (between HP and MP) if between 0 and 1, LPMP (between LP and MP) if between −1 and 0, and BLP (below LP) if less than −1. Genes where the hybrid expression level was significantly different from MP, but not from both HP and LP, were placed in an unclassified category (UC).

### Identification of genes with TEs

The annotated TEs present in the Arabidopsis Col genome were downloaded from TAIR [58], and compared to the annotated gene models using an in-house Perl script. If an annotated TE overlaps an annotated gene, defined as from the transcriptional start site to the end of 3’ UTR, including UTR, exon and intron, this gene was called as “containing a TE within the gene boundaries”. The identification of TEs upstream or downstream of a gene was performed in the same way.

### Availability of supporting data

The RNA and small RNA sequencing data for the two accessions and their two reciprocal hybrids are available at NCBI under accession number SRP058781.

## Additional files

Additional file 1:
**Sampled tissues in this study.** The four youngest leaves with visible petioles when the plants start to bolt were harvested, pooled and used for RNA extraction as one biological replicate. This figure shows the tissues sampled for each replicate. A total of four biological replicates, each a pool of four leaves (RNA) or the remaining rosette (DNA) were collected from four individuals of each of the two parental accessions and the reciprocal hybrids. Additional file 2:
**Small RNA and mRNA mapping statistics.**
Additional file 3:
**Small RNA and mRNA datasets used in this study.**
Additional file 4:
**Gene ontology terms that are enriched in groups of genes with different inheritance patterns.**
Additional file 5:
**The distance of TEs from a gene and its effect on mRNA inheritance.** “Body” is the body of the gene, defined as the interval between transcriptional start site and the end of the 3’ UTR, including UTRs, introns and exons. Additional file 6:
**Spearman correlation between small RNA level and mRNA level.**
Additional file 7:
**Replication of our results in a second cross of two Arabidopsis genotypes.** (A) Association between small RNA level and mRNA level in Arabidopsis accession C24. (B) Percentage of sssRNA in two groups of genes (refer to A for groups). (C) TE composition in two groups of genes (refer to A for groups). (D) Percentage of sssRNA for genes with different d/a value. d/a (dominance/additive) was used as a surrogate for inheritance pattern. A greater positive value resembles high-parent inheritance (HP), a value around 0 resembles mid-parent inheritance (MP), and a smaller negative value resembles low-parent inheritance (LP). (E) DNA methylation pattern in genes with different percentage of sssRNA. (F) DNA methylation pattern in genes showing different d/a value. Additional file 8:
**Nucleotide distribution of small RNA from different strands.** (A) Nucleotide composition per base in sense strand small RNA matching genes where small RNAs are predominantly (>90 %) from the sense strand. (B) Nucleotide composition in sense strand small RNA for genes where small RNAs match both strands. (C) Nucleotide composition in antisense strand small RNA for genes where small RNAs match both strands. Additional file 9:
**Distribution of small RNAs across predicted transcripts for genes with high expression levels.** (A) Line plot to show the combined distribution of small RNAs for all genes with high expression (>1000 normalized reads, see Fig. 3). Each transcript was divided into 100 equally sized windows by length, and the coverage of the nucleotides in each window as a percentage of the total coverage for the transcript was plotted. (B-D) Three examples of small RNA coverage of specific transcripts that show sense strand only small RNA.

## Abbreviations

AHP: Above HP
BLP: Below LP
HP: High-parent
HPMP: Between HP and MP
LP: Low-parent
LPMP: Between LP and MP
MP: Mid-parent
sRNA: small RNA
siRNA: Small interfering RNA
sssRNA: Sense strand small RNA
TE: Transposable element
UC: Unclassified
