# Supplementary figures and images for: Transposable elements, mRNA expression level and strand-specificity of small RNAs are associated with non-additive inheritance of gene expression in hybrid plants
Source: BMC Plant Biol. 2015 Jul 3;15:168. doi: 10.1186/s12870-015-0549-7 (PMC4490736; doi:10.1186/s12870-015-0549-7)

## Slide 1
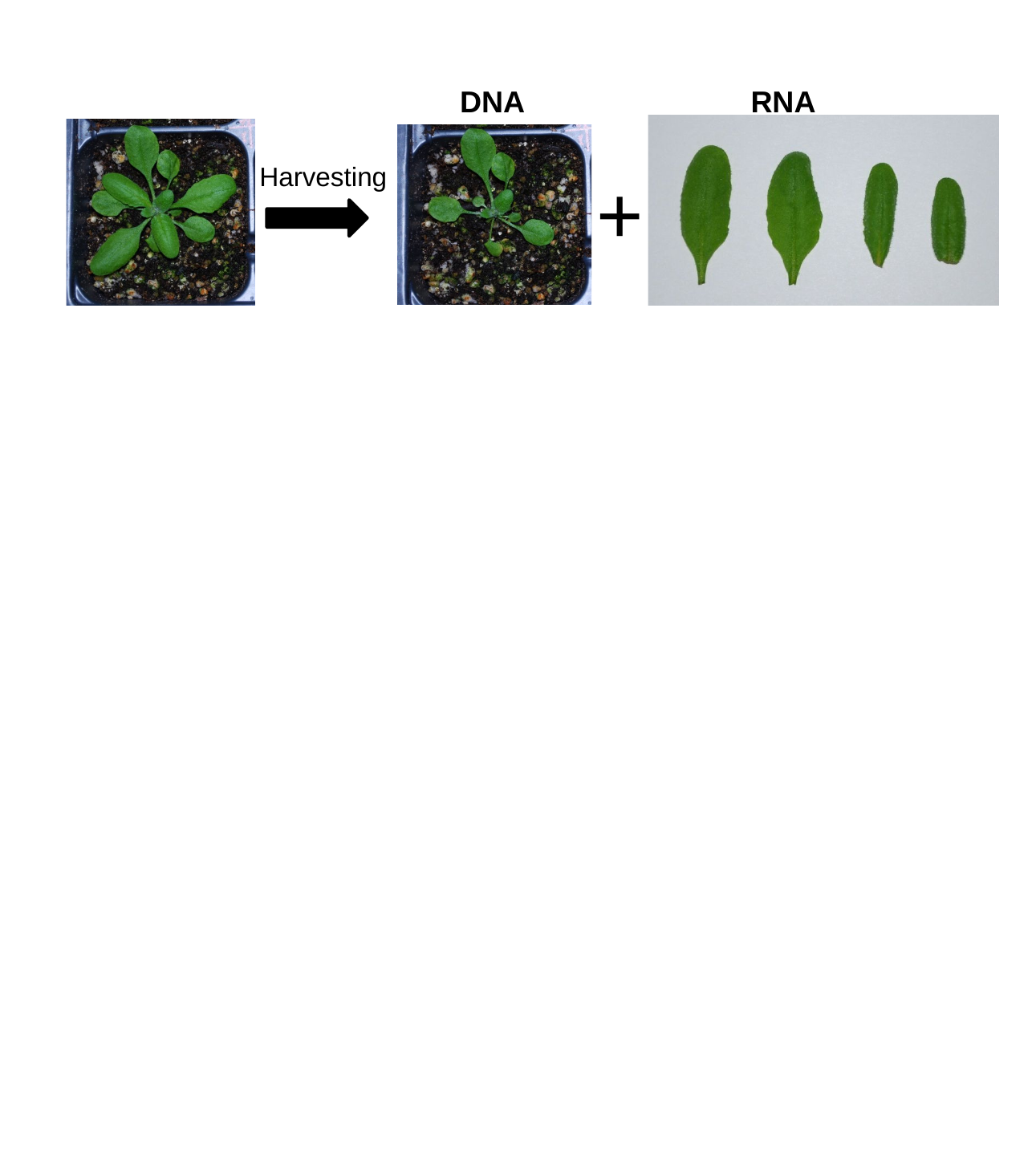

DNA
RNA
Harvesting
+

Supplement: Additional file 1: — Sampled tissues in this study. The four youngest leaves with visible petioles when the plants start to bolt were harvested, pooled and used for RNA extraction as one biological replicate. This figure shows the tissues sampled for each replicate. A total of four biological replicates, each a pool of four leaves (RNA) or the remaining rosette (DNA) were collected from four individuals of each of the two parental accessions and the reciprocal hybrids. [file 12870_2015_549_MOESM1_ESM.pptx]

## Slide 1
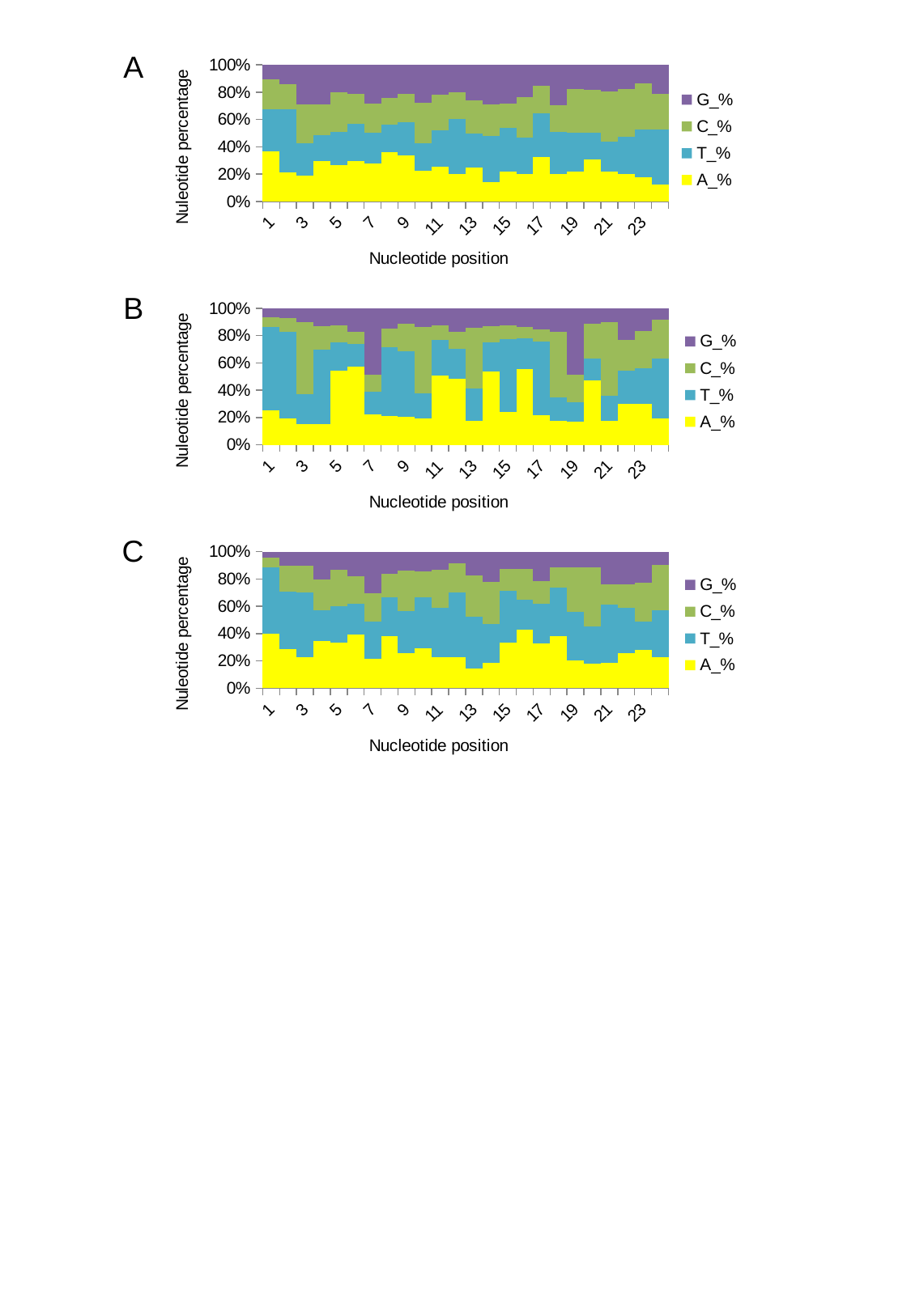

A
### Chart
| Category | A_% | T_% | C_% | G_% |
|---|---|---|---|---|B
### Chart
| Category | A_% | T_% | C_% | G_% |
|---|---|---|---|---|C
### Chart
| Category | A_% | T_% | C_% | G_% |
|---|---|---|---|---|

Supplement: Additional file 8: — Nucleotide distribution of small RNA from different strands. (A) Nucleotide composition per base in sense strand small RNA matching genes where small RNAs are predominantly (>90 %) from the sense strand. (B) Nucleotide composition in sense strand small RNA for genes where small RNAs match both strands. (C) Nucleotide composition in antisense strand small RNA for genes where small RNAs match both strands. [file 12870_2015_549_MOESM8_ESM.pptx]
