# Supplementary material for: Transposable elements, mRNA expression level and strand-specificity of small RNAs are associated with non-additive inheritance of gene expression in hybrid plants
Source: BMC Plant Biol. 2015 Jul 3;15:168. doi: 10.1186/s12870-015-0549-7 (PMC4490736; doi:10.1186/s12870-015-0549-7)
Supplement: Additional file 5: — The distance of TEs from a gene and its effect on mRNA inheritance. “Body” is the body of the gene, defined as the interval between transcriptional start site and the end of the 3’ UTR, including UTRs, introns and exons. [file 12870_2015_549_MOESM5_ESM.pptx]

## Slide 1
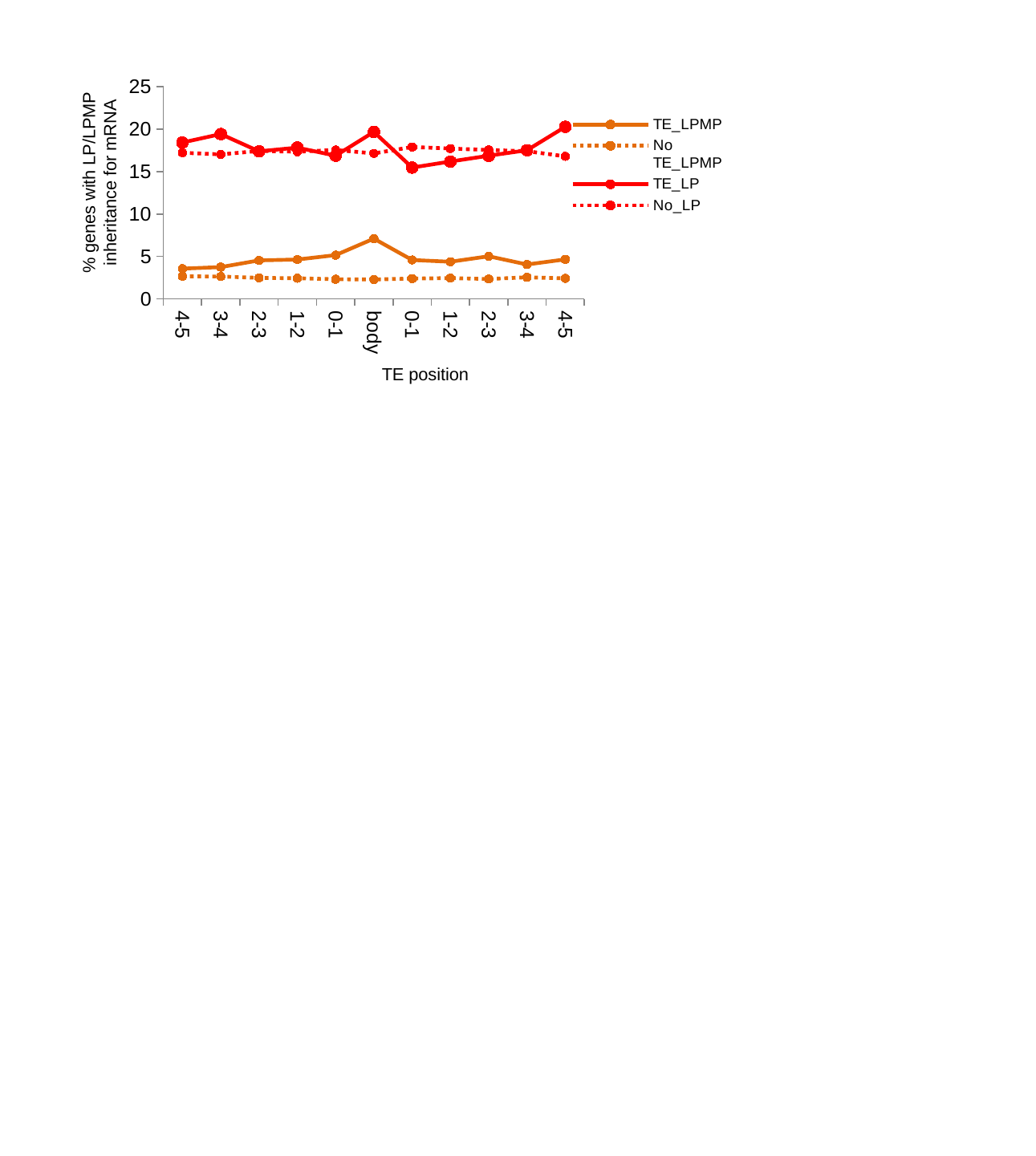

### Chart
| Category | | | | |
|---|---|---|---|---|
| 4-5k_up | 3.560209424083767 | 2.659232253913788 | 18.429319371727722 | 17.22067338623203 |
| 3-4k_up | 3.735325506937035 | 2.627643665883358 | 19.423692636072563 | 17.026276436658833 |
| 2-3k_up | 4.535637149028077 | 2.47229326513214 | 17.38660907127428 | 17.43393009377664 |
| 1-2k_up | 4.6324269889224565 | 2.4216216216216218 | 17.82477341389729 | 17.340540540540523 |
| 0-1k_up | 5.151515151515148 | 2.31201382886776 | 16.86868686868687 | 17.545375972342253 |
| body | 7.09677419354839 | 2.28091236494598 | 19.677419354838708 | 17.146858743497422 |
| 0-1k_down | 4.5787545787545785 | 2.3862129916040633 | 15.476190476190473 | 17.89659743703049 |
| 1-2k_down | 4.372093023255814 | 2.443319392471937 | 16.186046511627886 | 17.719568567026194 |
| 2-3k_down | 5.020080321285138 | 2.3366508005192532 | 16.867469879518072 | 17.54651665945478 |
| 3-4k_down | 4.042348411934556 | 2.533304214894082 | 17.51684311838306 | 17.405547062677428 |
| 4-5k_down | 4.648862512363996 | 2.4093770349468198 | 20.276953511374877 | 16.80052094638592 |% genes with LP/LPMP inheritance for mRNA
TE position
