# Supplementary material for: Transposable elements, mRNA expression level and strand-specificity of small RNAs are associated with non-additive inheritance of gene expression in hybrid plants
Source: BMC Plant Biol. 2015 Jul 3;15:168. doi: 10.1186/s12870-015-0549-7 (PMC4490736; doi:10.1186/s12870-015-0549-7)
Supplement: Additional file 7: — Replication of our results in a second cross of two Arabidopsis genotypes. (A) Association between small RNA level and mRNA level in Arabidopsis accession C24. (B) Percentage of sssRNA in two groups of genes (refer to A for groups). (C) TE composition in two groups of genes (refer to A for groups). (D) Percentage of sssRNA for genes with different d/a value. d/a (dominance/additive) was used as a surrogate for inheritance pattern. A greater positive value resembles high-parent inheritance (HP), a value around 0 resembles mid-parent inheritance (MP), and a smaller negative value resembles low-parent inheritance (LP). (E) DNA methylation pattern in genes with different percentage of sssRNA. (F) DNA methylation pattern in genes showing different d/a value. [file 12870_2015_549_MOESM7_ESM.pptx]

## Slide 1
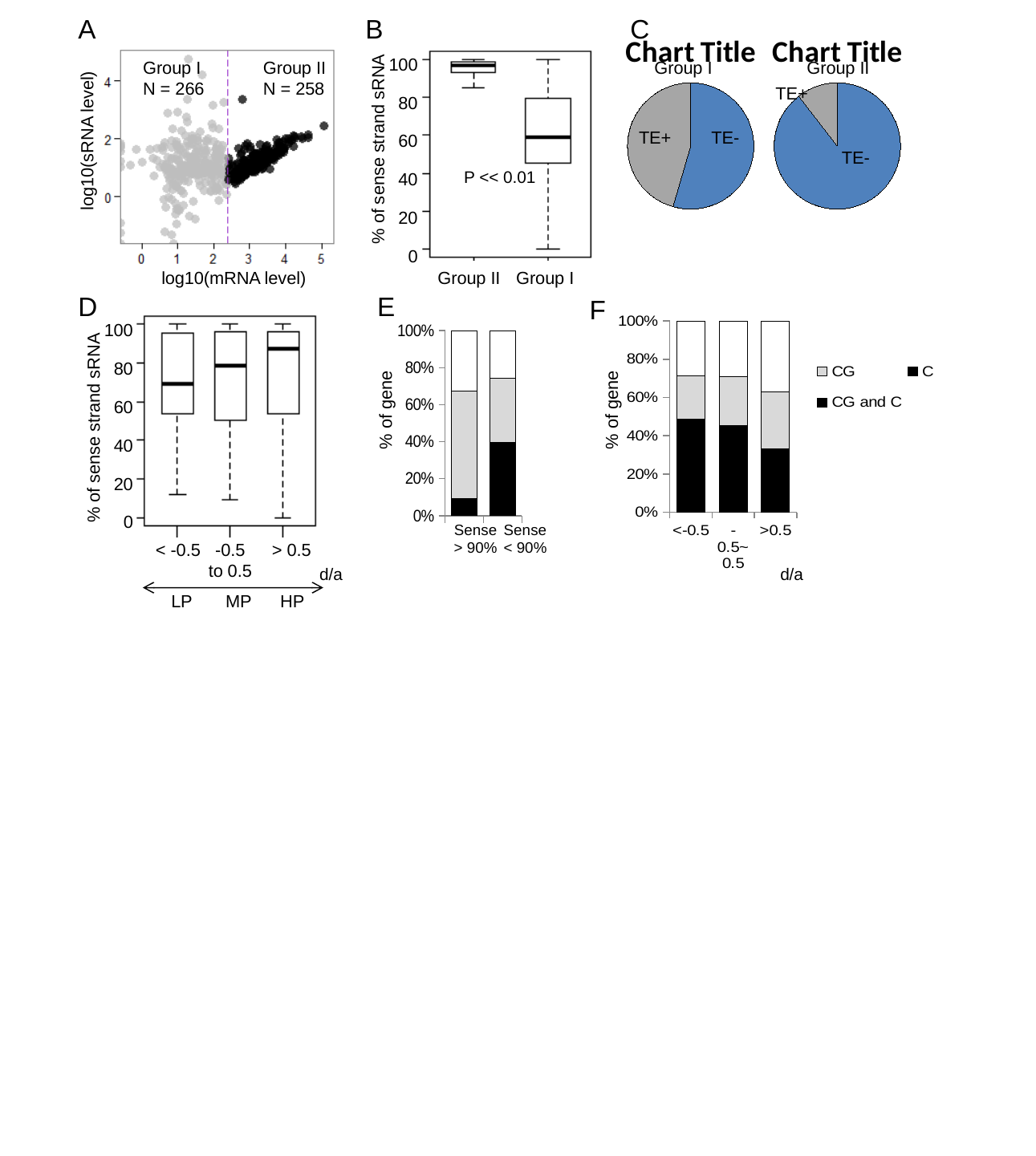

A
B
C
### Chart:
| Category | |
|---|---|
### Chart:
| Category | |
|---|---|100
80
60
40
20
0
Group I
N = 266
Group II
N = 258
Group I
Group II
TE+
TE+
TE-
log10(sRNA level)
% of sense strand sRNA
TE-
P << 0.01
log10(mRNA level)
Group II
Group I
D
E
F
100
80
60
40
20
0
### Chart
| Category | CG and C | C | CG | No methylation |
|---|---|---|---|---|
| <-0.5 | 17.14285714285723 | 31.428571428571427 | 22.85714285714283 | 28.571428571428573 |
| -0.5~0.5 | 3.6363636363636327 | 41.81818181818186 | 25.454545454545453 | 29.090909090909086 |
| >0.5 | 3.92156862745098 | 29.41176470588235 | 29.41176470588235 | 37.254901960784295 |
### Chart
| Category | CG and C | C | CG | No methylation |
|---|---|---|---|---|
| ss sRNA ≥ 90% | 1.1278195488721798 | 8.270676691729323 | 57.89473684210549 | 32.706766917293194 |
| other | 16.14173228346449 | 23.228346456692886 | 35.039370078740156 | 25.590551181102363 |% of gene
% of gene
% of sense strand sRNA
Sense > 90%
Sense < 90%
< -0.5
-0.5 to 0.5
> 0.5
d/a
d/a
LP MP HP
