# Supplementary material for: Transposable elements, mRNA expression level and strand-specificity of small RNAs are associated with non-additive inheritance of gene expression in hybrid plants
Source: BMC Plant Biol. 2015 Jul 3;15:168. doi: 10.1186/s12870-015-0549-7 (PMC4490736; doi:10.1186/s12870-015-0549-7)
Supplement: Additional file 9: — Distribution of small RNAs across predicted transcripts for genes with high expression levels. (A) Line plot to show the combined distribution of small RNAs for all genes with high expression (>1000 normalized reads, see Fig. 3). Each transcript was divided into 100 equally sized windows by length, and the coverage of the nucleotides in each window as a percentage of the total coverage for the transcript was plotted. (B-D) Three examples of small RNA coverage of specific transcripts that show sense strand only small RNA. [file 12870_2015_549_MOESM9_ESM.pptx]

## Slide 1
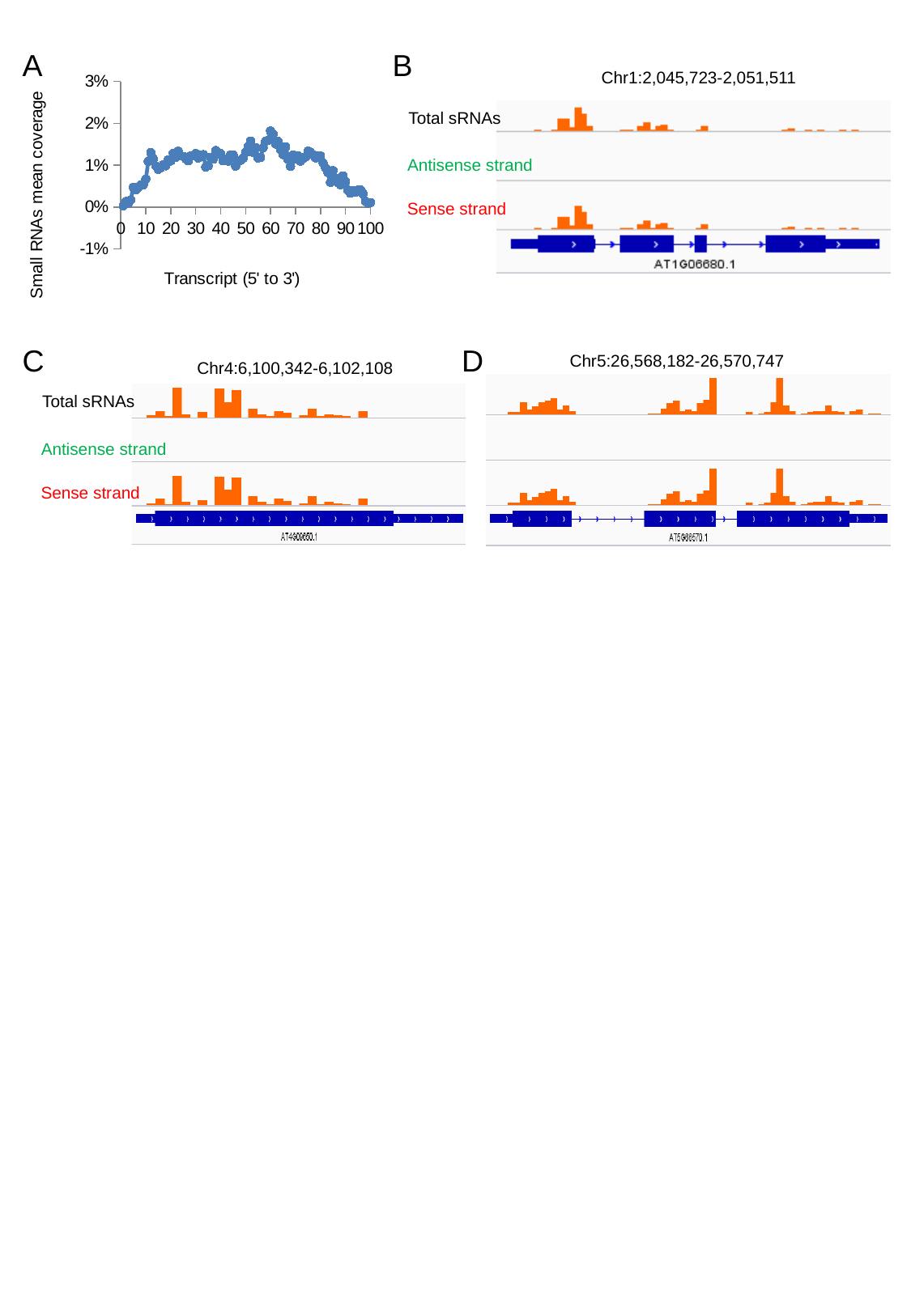

### Chart
| Category | mean coverage |
|---|---|A
B
Chr1:2,045,723-2,051,511
Total sRNAs
Antisense strand
Sense strand
C
D
Chr5:26,568,182-26,570,747
Chr4:6,100,342-6,102,108
Total sRNAs
Antisense strand
Sense strand
